# A Metastable Equilibrium Model for the Relative Abundances of Microbial Phyla in a Hot Spring

**DOI:** 10.1371/journal.pone.0072395

**Published:** 2013-09-02

**Authors:** Jeffrey M. Dick, Everett L. Shock

**Affiliations:** 1 Department of Chemistry and Department of Applied Geology, Curtin University, Perth, Western Australia, Australia; 2 School of Earth and Space Exploration and Department of Chemistry and Biochemistry, Arizona State University, Tempe, Arizona, United States of America; J. Craig Venter Institute, United States of America

## Abstract

Many studies link the compositions of microbial communities to their environments, but the energetics of organism-specific biomass synthesis as a function of geochemical variables have rarely been assessed. We describe a thermodynamic model that integrates geochemical and metagenomic data for biofilms sampled at five sites along a thermal and chemical gradient in the outflow channel of the hot spring known as “Bison Pool” in Yellowstone National Park. The relative abundances of major phyla in individual communities sampled along the outflow channel are modeled by computing metastable equilibrium among model proteins with amino acid compositions derived from metagenomic sequences. Geochemical conditions are represented by temperature and activities of basis species, including pH and oxidation-reduction potential quantified as the activity of dissolved hydrogen. By adjusting the activity of hydrogen, the model can be tuned to closely approximate the relative abundances of the phyla observed in the community profiles generated from BLAST assignments. The findings reveal an inverse relationship between the energy demand to form the proteins at equal thermodynamic activities and the abundance of phyla in the community. The distance from metastable equilibrium of the communities, assessed using an equation derived from energetic considerations that is also consistent with the information-theoretic entropy change, decreases along the outflow channel. Specific divergences from metastable equilibrium, such as an underprediction of the relative abundances of phototrophic organisms at lower temperatures, can be explained by considering additional sources of energy and/or differences in growth efficiency. Although the metabolisms used by many members of these communities are driven by chemical disequilibria, the results support the possibility that higher-level patterns of chemotrophic microbial ecosystems are shaped by metastable equilibrium states that depend on both the composition of biomass and the environmental conditions.

## Introduction

The structures of microbial communities emerge from a combination of environmental, ecological, and evolutionary interactions. Gradients of temperature are apparent in hot springs in Yellowstone National Park, and chemical properties such as pH, oxidation-reduction potential, and concentrations of dissolved sulfide and inorganic carbon also show great variation among sites, providing the foundation for delineating different possible chemotrophic metabolisms that may take advantage of the disequilibrium among inorganic chemical species [1]. In the same places where the chemical changes are apparent, there are gradations between major taxonomic groups. An example is the transition between chemotrophy and the onset of phototrophic metabolisms at lower temperatures [2]–[5]. This transition is sometimes referred to as the “photosynthetic fringe” [2] and is regarded as an ecotone [4], i.e. a transition between ecosystems with different metabolic and taxonomic characteristics.

The relative contributions of temperature, pH and sulfide to the limits of photosynthesis in Yellowstone have been quantified using statistical model selection techniques and generalized additive models [6], [7]. Larger-scale geochemical variation across hot springs in Yellowstone can also be correlated with phylogenetic trends determined from 16S RNA or metagenomic sequencing, for example using ordination methods such as principal components analysis [8].

Considering microbial communities in general, “bioclimatic models” have been proposed to assess the correlations between relative abundances and environmental variables [9]. Of these, artificial neural network models are noteworthy because they include terms describing interactions among taxonomic groups as well as with the environment [9], [10]. If the dynamics of community assembly (i.e., birth, death and immigration) are expressed as stochastic processes, another possibility is to use neutral community assembly to predict species abundance distributions [11], [12]. Neutral models invoke a mechanism for assembling microbial communities in which the probability for replacement of a cell is independent of species identity; these dynamics permit coexistence of species with varying competitive advantages [11].

Commonly, ecological models that predict relative abundances are based on mechanisms inherent in community assembly. A chemical equilibrium model, in contrast, describes only a single state of the system (an energy minimum), yet generates predictions linking the chemical conditions and the relative abundances of (bio)chemical species. Precedents exist for using energy minimization to describe certain aspects of biological systems; for example, analogies have been drawn between processes leading to increased fitness and energy minimization in chemical and physical systems [13]. Arguments for thermal equilibrium (maximum entropy) have been put forward to explain sequence diversity of proteins [14], but these were based on information-theoretic, not chemical, considerations. At the level of cells, equilibrium models to describe molecular binding lead to quantitative predictions and falsifiable hypotheses; failures of data to fit simple models provide evidence for energy coupling between processes [15]. The wide applicability of equilibrium models for binding was proposed to depend on the separation of time scales between the fast binding processes, taken to approach equilibrium, and the non-equilibrium changes in the cell such as concentrations of ions or gene transcripts [15]. A separation of time scales is also inherent in the present study, in that the equilibrium models investigated relate to long time scales (e.g. differences in the biomolecular compositions of organisms, which arise through evolution) compared to metabolism and other processes in single cells that proceed in non-equilibrium states.

Investigating the applicability of an equilibrium framework requires building quantitative models based on thermodynamic and compositional data; these challenges have made it so that possible implications for using predictions from equilibrium models to better understand environmental influences on microbial communities remain relatively unexplored. A metastable equilibrium model can be used to generate predictions connected with the hypothesis that complex interactions in ecology and evolution have a tendency to result in lowering of Gibbs energy of the ecosystem. A quantitative description of the local energy minimum leads to comparisons with data that may not fit the model and therefore can help to identify additional energetic contributions. These comparisons are attainable by first constructing a metastable equilibrium model that integrates parameters of the geochemical environment, biomolecular composition and relative abundances of coexisting microbial taxa. In this study, taxonomic classification at the phylum level is chosen in order to provide an overview of the entire community; but in the future it may be possible to develop comparisons at other taxonomic levels.

Here, we propose an application of a metastable equilibrium model for the relative abundances of microbial phyla (MEM-RAMP). A BLAST-based taxonomic assignment is used to classify the metagenomic sequences, and from the counts of assigned sequences to estimate the relative abundances of the phyla, referred to below as the observed abundances. The amino acid composition of a single model protein for each of the major phyla is constructed by binning metagenomic sequences using the BLAST assignments. The inputs to the model are then the geochemical conditions, including temperature, pH and oxidation-reduction potential, and amino acid compositions of the model proteins. The algorithms used in the model are based on group additivity for estimating the standard thermodynamic properties of proteins [16], and a metastable equilibrium calculation that can be derived from the Boltzmann distribution, as described in more detail below. The outputs of the model are the relative abundances of the model proteins, thereby constituting independent predictions of the relative abundances of the microbial taxa, referred to below as the metastable equilibrium or calculated abundances.

The model is a *metastable* equilibrium model because only the relative abundances of model proteins are calculated based on equalization of chemical affinities (see Methods for details); absolute abundances of proteins in equilibrium with other biomolecules or inorganic species are not considered. The candidate proteins used to model a community at a specific location are drawn from the metagenomic sequences and major phyla observed to be present at that location, not from the entirety of sequences and organisms in the hot spring. The outputs of the model can be compared with observed abundance distributions in order to optimize the geochemical parameters in the model. All of the geochemical parameters in the model are measurable and therefore the optimized model represents an integrative set of predictions based on observations from both geochemistry and microbial ecology, which is, nevertheless, made independently of any statement about mechanism or interaction among species. By comparing the model with observations, communities can be shown to have differing degrees of equilibration, and occurrences of taxa whose relative abundances diverge from metastable equilibrium lead to identification of additional inputs or outputs of energy.

## Results and Discussion

The model system studied here is the hot spring known as “Bison Pool” in Yellowstone National Park. “Bison Pool” is an unofficial name for the “fourth unnamed spring in Sentinel Meadow” identified in a study of the geochemical energy supply to hydrothermal ecosystems [1]. Over a distance of ∼20 m, the outflow channel of this hot spring is characterized by significant cooling (from boiling to less than 60°C) and increases in pH and oxidation-reduction (redox) potential. Chemosynthetic microbes, in some cases forming streamer biofilm communities [4], are common near the source of the hot spring, while mixed photosynthetic-chemosynthetic communities become prevalent at lower temperature. The gradients of temperature, pH and redox conditions along the outflow channel of the hot spring coincide with changes in composition of microbial communities that are apparent from metagenomic sequencing. Recent publications [4], [17], [18] provide detailed descriptions of the geochemistry and microbial communities of Bison Pool.

In the calculations described below, we first obtain a model protein for each phylum with an amino acid composition derived from metagenomic sequences. A stoichiometric analysis shows that the chemical compositions of these model proteins increase in average oxidation state of carbon down the outflow channel, but there is also a decrease in the range of oxidation states represented. Second, the relative abundances of the model proteins in metastable equilibrium are calculated as functions of a redox variable, 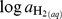, but temperature and chemical activities of basis species including pH are taken from field measurements. A major result is that the model is conformable to the observed relative abundances of phyla derived from a BLAST classification.

Finally, the values of 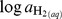 (the thermodynamic activity of dissolved 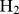) are optimized in order to minimize the energetic difference between the calculated and observed community profiles. The optimal values of activity of hydrogen decrease with distance down the outflow channel, in parallel with field-based redox measurements and with a previous model for the relative stabilities of proteins among sites [19]. The deviations between the equilibrium and observed abundances may be explained by energetic contributions from specific metabolisms. For example, low computed relative abundances of Chloroflexi and Cyanobacteria are consistent with additional energy input by phototrophy. The comparisons reveal an apparent minimization of Gibbs energy that has proceeded to a greater extent at the lower temperatures of the hot-spring ecosystem.

### Metagenomic Community Profile and Model Proteins

The metagenomic sequences were derived from biofilm samples at five sites along the outflow channel of Bison Pool [4], [17]–[19], representing two high-temperature chemotrophic communities (sites 1 and 2), a transition zone or ecotone (site 3) [4], and two lower-temperature phototrophic-chemotrophic communities (sites 4 and 5). The metagenomically derived protein sequences were classified using BLAST [20] against the NCBI RefSeq database [21], release 57; the output files are provided in Dataset S1. The major phyla are taken to be those that constitute at least 3% of the BLAST hits at a given location (see Methods for details). The number of inferred protein sequences at each of the five sampling sites in Bison Pool is listed in Table 1, and the BLAST-generated community profile is summarized in Tables 2 and 3.

**Table 1:** Overview of BLAST results, field measurements of temperature and pH, and model values of 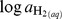.

						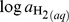	
Site^a^	Proteins^b^	BLAST hits^c^	Major phyla^d^	*T*, °C^e^	pH^e^	Eq. 2^f^	optimum^g^
1 (N)	40360	32602	5 (28901)	93.3	7.350	−4.00	−3.38
2 (S)	50497	37333	7 (31786)	79.4	7.678	−5.04	−4.14
3 (R)	43250	31886	7 (26163)	67.5	7.933	−5.94	−5.66
4 (Q)	83790	66490	7 (58073)	65.3	7.995	−6.10	−7.47
5 (P)	74082	57344	7 (47744)	57.1	8.257	−6.72	−10.02

a The number of the sampling site in the hot spring is given, together with the original letter codes used in the field to identify the samples.

b The number of inferred protein sequences in the metagenome (from JGI annotations), which in general do not correspond to complete protein sequences.

c The number of hits using protein BLAST to the microbial proteins in the RefSeq database version 57.

d Numbers of major phyla (i.e. those making up at least 3% of the total number of BLAST hits in a given site) and, in parentheses, hits that are assigned to a major phylum.

e Field-based measurements in the hot spring [1], [19].

f Gradient model; calculated using Eq. 2 [19] and the values of temperature shown in this table.

g Community model; these values minimize the difference in Gibbs energy between assemblages having the metastable equilibrium and observed relative abundances of phyla (Eq. 16).

**Table 2:** Summary of major phyla at each location in the hot spring.

Phylum^a^	Sequences	Representative Species (%)^b^	Phylum^a^	Sequences	Representative Species (%)^b^
Site 1 (N)			Site 4 (Q)		
Aquificae	15878	1 (39.2)	Chloroflexi	19149	13 (63.8)
Crenarchaeota	5712	2 (18.6)	Cyanobacteria	15593	14 (58.4)
Proteobacteria	4462	3 (16.0)	Proteobacteria	8135	3 (18.9)
Dein.-Thermus	1668	4 (75.5)	Acidobacteria	5209	15 (88.3)
Firmicutes	1181	5 (3.2)	Firmicutes	4474	16 (2.8)
			Bacteroidetes	3036	10 (8.3)
Site 2 (S)			Chlorobi	2477	17 (83)
Aquificae	9549	6 (38.6)			
Crenarchaeota	7646	2 (14.6)	Site 5 (P)		
Proteobacteria	6195	3 (9.6)	Chloroflexi	17557	13 (43.8)
Firmicutes	3872	7 (10.4)	Proteobacteria	8385	3 (19.0)
Dein.-Thermus	1986	4 (70.1)	Cyanobacteria	8158	12 (50.8)
Euryarchaeota	1301	8 (5.5)	Firmicutes	5590	16 (3.3)
Chloroflexi	1237	9 (17.9)	Acidobacteria	3500	15 (83.2)
			Bacteroidetes	2373	10 (13.2)
Site 3 (R)			Dein.-Thermus	2181	4 (38.2)
Dein.-Thermus	8493	4 (73.3)			
Firmicutes	5406	7 (13.5)			
Proteobacteria	3078	3 (5.2)			
Aquificae	2935	6 (36.4)			
Bacteroidetes	2624	10 (19.5)			
Chloroflexi	2493	11 (40.4)			
Cyanobacteria	1134	12 (5.6)			

a The names of the major phyla having sequences making up at least 3% of the total number of BLAST hits in any location.

b The species with the greatest number of BLAST hits (percentage shown in parentheses) in each phylum. The numbered species are listed in Table 3.

**Table 3:** Representative species having greatest number of BLAST hits for major phyla at different sites in the hot spring.

Number	Name		Number	Name
1	*Thermocrinis albus*		10	*Rhodothermus marinus*
2	*Pyrobaculum* sp. 1860		11	*Thermomicrobium roseum*
3	*Enterobacter cloacae*		12	*Synechococcus* sp. JA-3-3Ab
4	*Thermus aquaticus*		13	*Roseiflexus* sp. RS-1
5	*Carboxydothermus*		14	*Synechococcus* sp. JA-2-3B’a(2-13)
	*hydrogenoformans*		15	“*Candidatus* Chloracidobacterium
6	*Hydrogenobacter thermophilus*			Thermophilum”
7	*Bacillus* sp. m3-13		16	*Thermincola potens*
8	*Ferroglobus placidus*		17	*Chloroherpeton thalassium*
9	*Anaerolinea thermophila*			

Numbers correspond to the species identifiers in Table 2.

In the BLAST-generated taxonomic profile, the only major phylum present at all sites is Proteobacteria. *Enterobacter cloacae*, commonly occurring in the human gut flora, is identified as the most highly-represented species in this phylum. There is a possibility that DNA from this organism was introduced at some point, either from the meadow surrounding the hot spring, or in the sample processing or sequencing pipeline, but also the possibility of artifacts in the BLAST assignments as a consequence of the relatively limited representation of hot-spring organisms in the RefSeq database. It is the classification of these sequences as coming from Proteobacteria, not the species-level assignment, that is used in the model.

The number of major phyla changes from 5 at site 1 to 7 at all other sites. An increase in number of phyla along the outflow channel is consistent with a previous analysis based on classification of metagenomic DNA sequences instead of proteins [18]. Also in line with that study and with analysis of small subunit RNA (16S) sequences [4], we find that the major bacterial group is Aquificae at sites 1 and 2, with Deinococcus-Thermus becoming most abundant at site 3. At sites 4 and 5 there is a greater dominance of Chloroflexi and Cyanobacteria, respectively, and at site 5 an abundance of Proteobacteria that exceeds that of Cyanobacteria, which is consistent with previously reported classifications [18]. The absence of Deinococcus-Thermus at site 4 in this analysis is consistent with the very low abundance found in [18]. Other notable similarities to [18] include the high proportion of Chloroflexi at site 3 that are classified as *Thermomicrobium roseum* and a change in dominant representation of Cyanobacteria from *Synechococcus* sp. strain B-prime to *Synechococcus* sp. strain A between sites 4 and 5 (Table 3).

The protein BLAST community profile indicates that Firmicutes are most abundant in the transition environment of site 3, which is consistent with previous studies at Bison Pool [4], [18]. Sequences classified as Acidobacteria at sites 4 and 5 belong mostly to “*Candidatus* Chloracidobacterium thermophilum”, with a genome sequence published in 2012 [22]. The rapidly developing nature of the reference genomic databases implies that at present there are significant limitations of using BLAST analysis to classify all the sequences in the metagenome. For classification at the phylum level at Bison Pool, BLAST is sufficient, while for more refined taxonomic assignments other binning approaches, such as binning based on tetranucleotide frequencies [18], should be considered.

The phylum distribution derived from BLAST was not corrected for sampling effects. Using more statistically rigorous markers such as single-copy protein coding genes [23], or 16S RNA sequences with copy number corrections [24] would improve the actual picture of the community. However, the similarities of the BLAST-derived community profile to previous studies suggests that it is suitable as a first-order estimate for the relative abundances of the phyla that can be used for testing the major questions posed in this study.

The BLAST profile also serves to identify sequences that are combined to generate the amino acid compositions of the model proteins. The quantity of each protein that is actually produced by the microbial communities is unknown. Therefore, we derived model protein compositions from an unweighted average of metagenomic sequences. The amino acid compositions and chemical formulas of the model proteins are listed in the files bison_protein.csv and protein_table.csv, respectively, in Dataset S2. Eventually, more robust metastable equilibrium models could be constructed that take account of the total composition of biomass (not only proteins) and Gibbs energies of cells. While there is progress in that direction, the attempts to date have been based on cellular compositions of common model organisms such as *Escherichia coli* and *Saccharomyces cerevisiae* [25], [26].

### Average Oxidation State of Carbon in Model Proteins

The average oxidation state of carbon (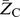) is a stoichiometric ratio that is useful for comparing the chemical compositions of sequences. It can be used to quickly assess the impact of changing environmental oxidation potential on the relative energy available for the overall reactions to form the proteins from inorganic constituents, although a thermodynamic assessment, described below, is needed to quantify the energies with regard to all environmental parameters, including temperature, oxidation potential, pH and concentrations of other chemical species. Average oxidation state of carbon can be calculated using [19]:
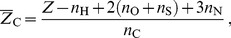
(1)where 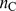, 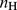, 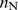, 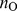 and 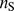 are the numbers of the indicated subscripted elements in the chemical formula of a protein or other chemical species, and 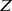 is the net charge of the chemical species. Note that protonation/deprotonation reactions have no net effect on 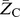 because the addition or removal of a proton contributes equally to 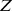 and 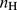.

**Figure 1:**
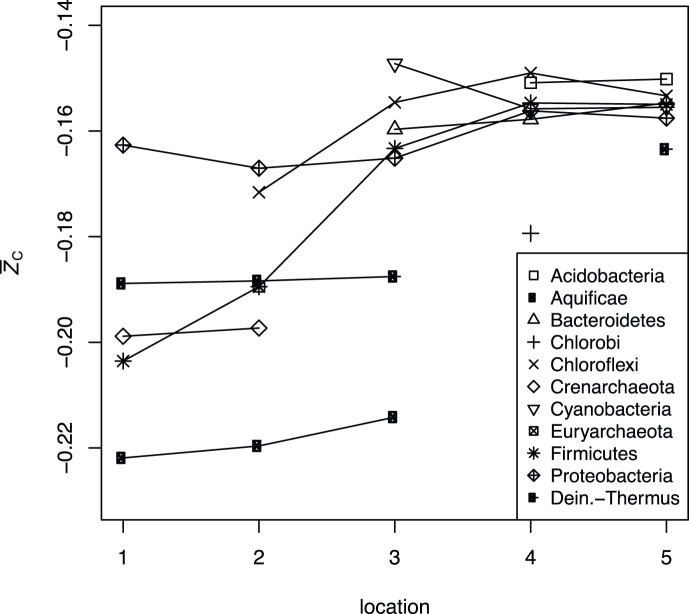
Average oxidation state of carbon (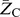), calculated using Eq.(1), of the model proteins for each of the major phyla listed in Table 2. Lines are drawn to connect the points for the same phylum identified at multiple sampling locations.

Along the length of the outflow channel of Bison Pool, there is a general increase in oxidation state of carbon in the model proteins (Fig. 1). The most reduced model proteins are those for Aquificae at sites 1 and 2, and the most oxidized model proteins are those for Cyanobacteria, Chloroflexi and Acidobacteria at sites 3, 4 and 5. The overall increase of the average oxidation state of carbon in model proteins for phyla shown in Fig. 1 is similar to that for model proteins representing shared functional annotations described in our previous study [19]. However, unlike the mostly sub-parallel increase of 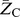 exhibited by different groups of model proteins representing shared functional annotations [19], model proteins for phyla show a wide range of 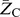 at sites 1, 2 and 3 that becomes much narrower at sites 4 and 5. This pattern indicates a greater chemical variation among model proteins for phyla at the higher temperatures.

Changes in chemical composition of hydrothermal fluids, in addition to temperature, can have major influences on the energy available to different microbial communities [1]. One possible environmental contribution to the trends appearing in Fig. 1 is that the input of oxygen to the ecosystem through mixing of the hot-spring fluid with the atmosphere and with more oxygenated groundwater, and perhaps also through diurnal production and consumption of 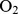 by photosynthetic organisms, results in more oxidizing conditions farther from the source of the hot spring. The magnitude of the effect of increasing oxidation potential of the fluid on the energy available for biomass synthesis is related to the chemical composition (stoichiometry) of the biomass itself. Increasing oxidation potential may provide more energy for the growth of chemotrophs generally, through the greater disequilibrium established with initially reduced species in the fluid. However, the chemical shift can be expected to have a relatively less favorable impact on the chemotrophic growth of Aquificae and other organisms that have relatively low-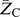 (reduced) biomass, compared to that of other organisms with more oxidized biomass. Other explanations may be needed for the observation that the source of the hot spring hosts phyla with a wide range of 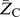, while the farther reaches exclude the most reduced model proteins. A theoretical explanation for this phenomenon is beyond the scope of this study, but as will be shown below, the compositional difference between model proteins is proportional to the range of their relative abundances in metastable equilibrium.

### Effects of Revised Methionine Sidechain Group on the “Gradient” Model

In a previous study [19], a comparison was made, among sampling locations, of the relative stabilities of model proteins representing various functional annotations in the Bison Pool metagenome. The results were used to formulate an operational linear correlation between temperature and redox conditions, represented by the logarithm of activity of hydrogen (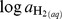). That equation, corrected for misplaced parentheses appearing in [19], is
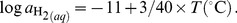
(2)


Eq. (2) is used here as a reference indicating the increase in oxidation potential down the channel of the hot spring, which is also reflected in field-based measurements of inorganic species [19]. Below, we refer to this reference line as the “gradient” model for redox conditions in the hot spring.

**Figure 2:**
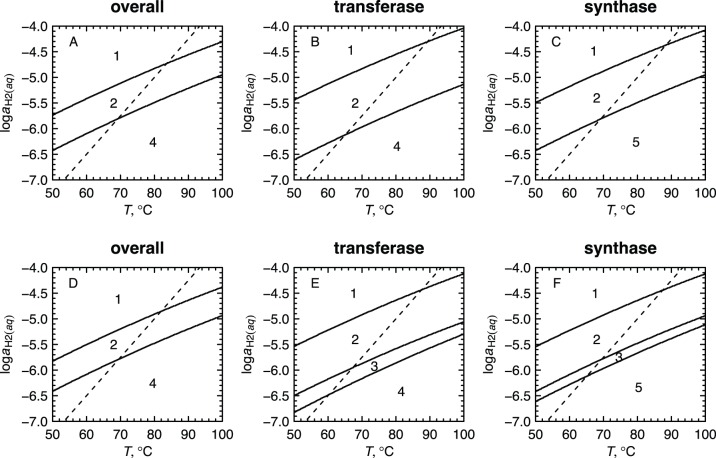
Predominance diagrams for selected groups of model proteins in the “gradient” model [19] as a function of 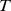 and 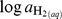. The model proteins have amino acid compositions taken from the bulk metagenome at each site (“overall”) or from sequences having the indicated functional annotations (“transferase”, “synthase”). Each plot depicts the stability relations among five model proteins, one from each site, indicated by the numbers. Numbers that do not appear in a given plot correspond to proteins that are less metastable than the others over the entire 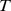-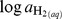 range that is shown. Plots (a–c) were constructed using the same set of thermodynamic data as used in [19] and closely reproduce the corresponding plots in Figs. 5b and 6 of that paper. Plots (d–f) were computed in this study using a revised, to a less negative value, standard Gibbs energy of formation of the methionine sidechain group taken from [27]. The less negative Gibbs energy of the methionine sidechain group tends to stabilize proteins that have a lower methionine content, resulting in the appearance of stability fields for site 3 in plots (e) and (f). The dashed lines in all figures indicate values of 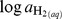 calculated using Eq. (2).

Revised values of the standard Gibbs energy and enthalpy of formation of the aqueous methionine sidechain group ([Met]) were recently published [27]. This revision, to more positive values of 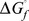 of aqueous methionine and [Met], improves the consistency between the calculated properties of methionine and the experimental enthalpy of combustion of the crystalline amino acid [27]. To assess the effect of this revision on the predicted relative stability limits of the proteins, a series of 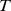-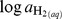 diagrams for model proteins in the gradient model [19] is shown in Fig. 2, calculated using both the old [16] and new [Met] group properties. The lines representing Eq. (2) cross the stability fields at similar positions in both cases. Therefore Eq. (2) is used as a reference line, without modification, but the updated properties of [Met] are used in the present study.

Using the updated thermodynamic properties of [Met], stability fields for site 3 appear in the diagrams for model proteins for transferase and synthase shown in Fig. 2, which was not the case previously [19]. Compared to the other sites, the proteins at site 3 have an overall lower abundance of methionine (overall percentage methionine equal to 1.98, 2.02, 1.90, 2.01 and 2.02 at sites 1–5, respectively, showing a significant depletion at site 3 compared to the variation among other sites), which is associated with a depletion of sulfur in model proteins at that site apparent in Fig. 3 of [19]. The appearance of stability fields for site 3 is therefore an expected result because a lower proportion of methionine is associated with a lower 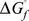 per residue using the updated properties of [Met]. Stability fields for model proteins from site 3 that were not apparent in [19] also appear for a couple of other functional categories (ATPase, protease; see Figure S1). The representation of greater numbers of locations in the hot spring on these diagrams lends support to the notion of a progression of local metastable equilibrium states that link the geochemical conditions and the amino acid compositions of proteins in the hot spring [19]. That outcome, however, is not a prerequisite for the community-based model described in the present study.

**Figure 3:**
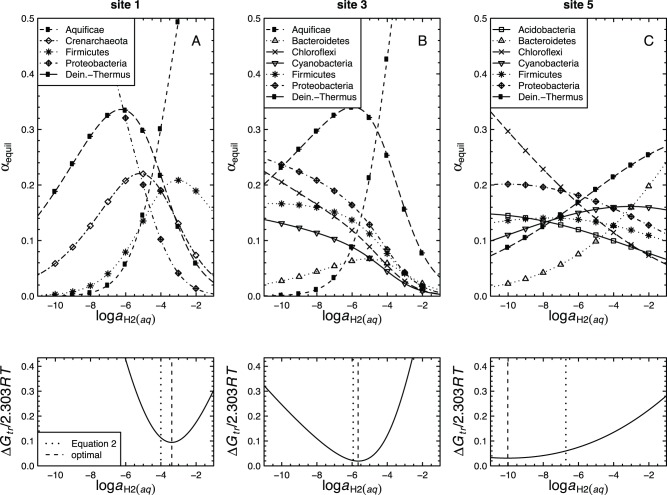
Calculated relative abundances of model proteins in metastable equilibrium (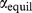) for the residue-normalized model proteins for the phyla listed in Table 2 for sites 1, 3 and 5 as functions of 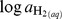. Values of 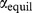 were calculated using Eqs. (3–7). The Gibbs energy of transformation (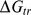; Eqs. 17–18), quantifying the difference between the calculated metastable equilibrium and observed relative abundances, which were generated by counting BLAST hits, is shown in the lower row of figures. The dotted and dashed vertical lines indicate, respectively, reference values of 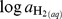 calculated as a linear function of temperature (Eq. 2), and optimal values of 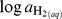, listed in Table 1, that minimize the value of 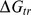.

### Optimizing the Metastable Equilibrium Model with a Redox Variable

Although values for 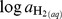 as a function of temperature were derived in the gradient model of our previous study (Eq. 2), here we use this redox variable to optimize the metastable equilibrium calculations for relative abundances of phyla. The metastable equilibrium abundances of the residues of the model proteins were calculated as functions of 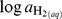 as described in the Methods. The results of models with variable 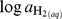 are shown in Fig. 3 (top row) for sites 1, 3 and 5. The relative abundances of the model proteins in metastable equilibrium strongly depend on the activity of hydrogen; in general, the computed relative abundances increase with increasing activity of hydrogen for those model proteins with more negative 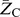 values, and vice versa. The computed relative abundances of the proteins change more rapidly with changing 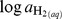 at site 1 than at sites 3 or 5. Note, for example, that while the range of 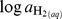 is the same in all plots shown in Fig. 3, the range of relative abundances extends to both higher (above 0.5) and lower (closer to 0) values in Fig. 3a (site 1) as compared to Fig. 3c (site 5). The greater degree of variation at site 1 is reflected in a more tightly constrained optimum for the value of 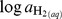 at this site (see discussion of 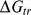 below). This behavior is consistent with the greater range of 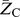 of model proteins at site 1 (Fig. 1), which is related to a greater range of stoichiometries of the relevant compositional variable (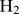) in the formation reactions of the model proteins. The differences in reaction stoichiometry in turn lead to relatively greater differences in chemical affinity (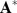 in Eqs. 6–7), at the limits of 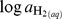 shown in Fig. 3, for the formation reactions of the proteins at site 1 compared to those at the other sites.

In the bottom row of Fig. 3, the Gibbs energy of transformation (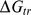), representing the energetic difference between metastable equilibrium and observed assemblages is plotted as a function of 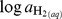. Optimal values of 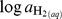, i.e. those that tune the model to best fit the observed relative abundances, are indicated by the minima in 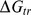. The optimal values of 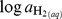 in the community model (this study) are different from those calculated using Eq. (2) derived using the gradient model of our earlier study [19], as can be seen by the offset between the dashed and dotted lines in the lower row of Fig. 3. The optimization was also performed for sites 2 and 4; the optimal values of 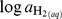 for all sites are listed in Table 1. The minimum values of 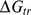 are lower at sites 3–5 than at sites 1 and 2, indicating a greater degree of equilibration for the lower-temperature sites. This tendency toward equilibration stands in contrast to the inorganic reactions supporting chemotrophic metabolism, many of which have higher affinities at lower temperatures (see Table S4 of [18]). The specific departures from metastable equilibrium in the present model may be explained by other energy inputs and/or differing growth efficiencies (see below).

**Figure 4:**
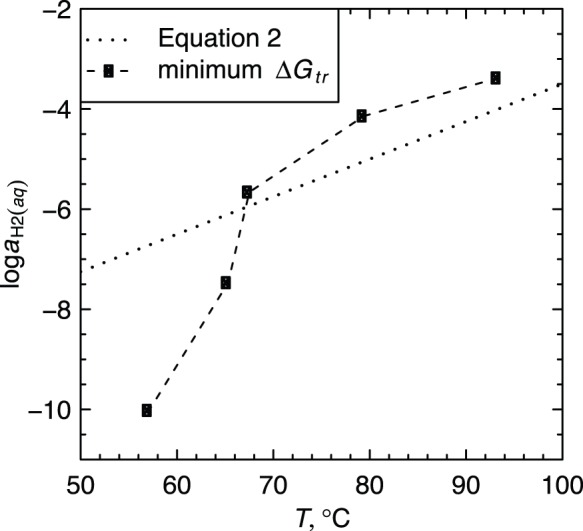
Values of 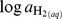 as a function of temperature calculated using two different models. The dotted line represents Eq. (2), which was used in [19] to calibrate a model for the relative chemical stabilities of model proteins among sites (“gradient” model). The points connected by the dashed line indicate optimal values of 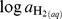 that were derived in the present study by minimization of the Gibbs energy of transformation between the metastable equilibrium relative abundances of model proteins for phyla and the BLAST-derived observed relative abundances of phyla.

As shown in Fig. 4, the calculations of optimal 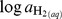 using the community model yield discrete points that have a similar overall slope at sites 1–3 to the gradient model of [19] represented by Eq. (2). The increase in 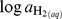 with temperature supports a less negative value of chemical affinity of protein synthesis, i.e. a reduced cost to form the proteins at site 1 (see Table 5 in [19]). In the community model (this study) there is a pronounced shift toward more oxidizing conditions, or lower 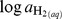, at lower temperatures compared to the gradient model. While it should be noted that the values obtained in this study are subject to greater uncertainty at lower temperatures due to the broader minimum in 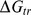 (see Fig. 3), the sharp decrease in 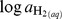 seen in Fig. 4 mirrors the increase in dissolved 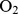 measured at sites 4 and 5 [18].

### Comparison of Metastable Equilibrium and Observed Relative Abundances

**Figure 5:**
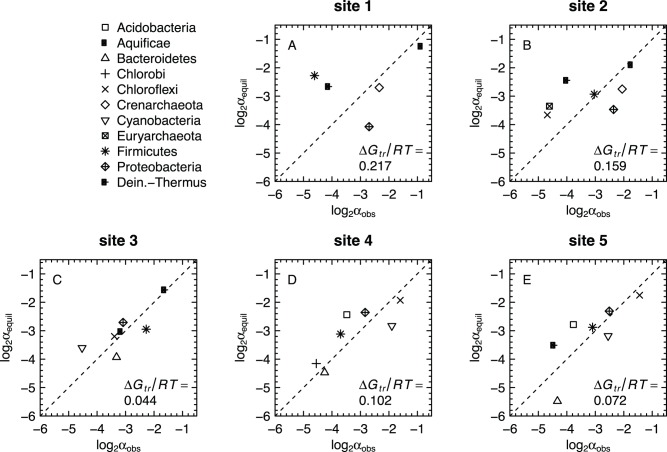
Comparison of calculated metastable equilibrium relative abundances of model protein residues (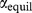) with observed relative abundances of phyla generated by counting BLAST hits (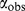). Values of 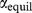 were calculated using values of 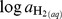, listed in Table 1, that minimize the Gibbs energy of transformation (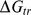) between the metastable equilibrium and observed distributions.

In Fig. 5 the metastable equilibrium degrees of formation of the residues are plotted against the observed BLAST profiles. The base 2 logarithms of the fractional relative abundances are used in Fig. 5 because the whole-number numeric gradations on the axes represent finer divisions (doublings) than the powers of ten that are associated with unit changes of decimal logarithms, which are more commonly used for chemical activities of species.

These figures can be interpreted in the following manner, using Fig. 5a as an example. Both methods show that Aquificae are the most abundant phylum at site 1, the source pool of the hot spring. The metastable equilibrium method yields a calculated abundance that is a factor of ca. 1.3 lower than the BLAST analysis. Other larger differences are apparent. As an example, the MEM-RAMP method yields a calculated abundance for Firmicutes that exceeds that from the BLAST analysis by a factor of 5. In contrast the BLAST analysis yields an abundance of Proteobacteria that exceeds the metastable equilibrium calculation by a factor of 2.6. As indicated by the values of 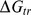, the differences between the BLAST and MEM-RAMP results are greatest for site 1, and are diminished at the other sites.

The relative abundances of model proteins shown in Fig. 5 represent a fit to the observed relative abundances by minimizing the Gibbs energy while solving for the relevant redox parameter. This redox parameter is 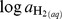, which is optimized to reduce the energetic difference between the metastable equilibrium and observed assemblages. Therefore, the metastable equilibrium relative abundances reflect geochemical constraints on the relative stabilities of biomolecules, and quantitatively represent the predictions of a hypothesis of an energy-minimizing link between the environment and the composition of the microbial communities. This hypothesis concerns only the environment and the composition of the communities, regardless of any specific mechanism for how they originated; but the specific patterns – both convergent and divergent – found in comparing the predictions and observations may have implications for process-oriented descriptions of the organisms or the community.

The optimized metastable equilibrium model is able to identify the phylum with the highest abundance at all sites. The model successfully reproduces the ranking of relative abundances of some of the lower-abundance phyla at sites 4 and 5, including Proteobacteria, Firmicutes and Bacteroidetes (Fig. 5d and e). Because the metastable equilibrium model can reproduce many aspects of the microbial community structure, it provides evidence for energy minimization of the protein biomass within geochemical constraints. Site 3 (Fig. 5c) exhibits the smallest Gibbs energy of transformation between metastable and observed assemblages at the optimal 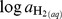, and can be said to be closest to metastable equilibrium. No implications are made by the metastable equilibrium model about how that energy minimization occurred, so the model offers a set of predictions that are independent of specific evolutionary mechanisms that could be at play.

Points near the dashed lines in Fig. 5 represent phyla that are close to metastable equilibrium. Points below the lines represent phyla that in reality are more abundant than predicted by the model. An increase in relative abundance, i.e. chemical activity, of a model protein leads to a lower (less positive) affinity of its formation reaction. Therefore, a higher-than-predicted observed abundance for a phylum can be interpreted to be caused by an additional input of energy – a contribution that is external to the energetics of the model reaction between basis species and model proteins. By adopting this interpretation we recognize that the communities may approach, but are not completely in, metastable equilibrium, owing to energetic contributions that can not be accommodated by the model.

The relative abundances of Chloroflexi and Cyanobacteria at sites 4 and 5 obtained by BLAST are greater than those predicted by the metastable equilibrium model. These differences between calculated and observed relative abundances are not as large as for some other phyla, particularly at sites 1 and 2, and Bacteroidetes at site 5, which are discussed below. A positive deviation of observed relative abundances requires that the reactions ultimately responsible for the formation of the model proteins for these two phyla from inorganic sources are driven by a greater supply of energy than is available to reactions to form other members of the community. This finding is consistent with a phototrophic source of energy for the biosynthesis of proteins in Chloroflexi and Cyanobacteria that exceeds the energy available to organisms with chemotrophic metabolisms. These results support the notion that the biomass (on a specific or per-residue basis) of these two phyla is more costly to produce, so sunlight may be used to drive the production of a greater quantity of biomass than would be obtained in a metastable equilibrium with the other phyla. A higher specific demand of energy for the production of biomass in phototrophs relative to chemotrophs might limit the range of environmental conditions suitable for photosynthesis, if they are controlled by the balance of supply and demand of energy [2].

Other underpredictions of relative abundance can be found at other sites, for example Proteobacteria at sites 1 and 2 and Bacteroidetes at site 5. These results may imply the existence of mechanisms other than photosynthesis to account for additional energy input. As with phototrophy, heterotrophic metabolisms may provide additional energy input to sustain biomass growth beyond the range predicted by the metastable equilibrium model. *Rhodothermus marinus* is a heterotroph [28] that is representative of Bacteroidetes at Bison Pool (Table 3). It should be noted that uncertainties in the observed relative abundances and the compositions of model proteins may also arise from addition of cells or DNA from sources outside the hot spring or errors in assigning compositions to the model proteins based on classifications of metagenomic sequences.

In contrast to the phototrophic phyla at lower temperatures, Deinococcus-Thermus is overpredicted by the model at high temperatures, which is most apparent at site 2 where the model predictions of its relative abundance are ∼3 times greater than in the BLAST profile (Fig. 5b). Therefore, in the thermodynamic model there is an excess of energy allocated to this phylum; its biomass has a lower specific energy compared to the other phyla. This divergence may be partially accounted for by a relatively high turnover rate of biomass. A high turnover rate would provide a way to expend the excess energy, by decreasing the overall efficiency of biomass production. Growth of *Thermus aquaticus* in continuous cultures in the laboratory was found to occur with lower efficiency than other thermophiles [29]. The actual mechanism for decreased growth efficiency in that case was not identified, but a partial decoupling between catabolism and anabolism is plausible [30].

**Figure 6:**
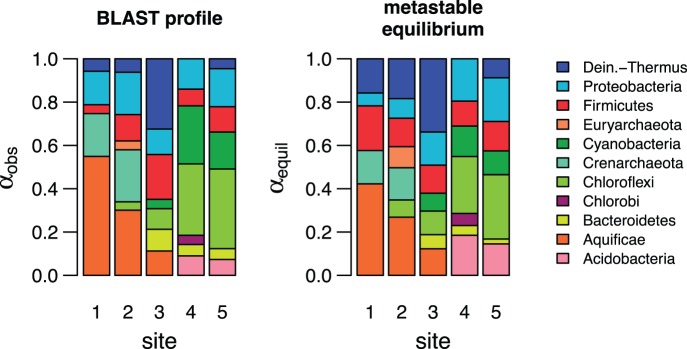
Community profiles showing abundances of phyla (a) observed in BLAST-based classification of metagenomically inferred protein sequences, and (b) calculated using the metastable equilibrium model.

The results of the metastable equilibrium model can also be visualized with the comparative bar charts in Fig. 6. It is important to note that the presence or absence of phyla is not a prediction made in this study; only their relative abundances can be predicted using the metastable equilibrium model. The compositions of proteins are derived from metagenomic sequences at individual sites, so that even in a given phylum there are variations in the compositions of model proteins (Fig. 1). The compositional variation in model proteins contributes to the ability of the proposed model to track the changing relative abundances of the phyla through the hot spring.

### Comparison with Other Models for Microbial Communities

Another model for equilibrium concentrations of microbial biomass (*E. coli* and *S. cerevisiae*) is the computation of “virtual equivalent concentration at equilibrium” (VECE) [26]. The major similarity with the present study lies in using taxon-specific chemical formulas and Gibbs energies. The results of the VECE model represent the stable equilibrium between biomass and the environment, corresponding to very low abundances of various cells. In contrast, in the current study the relative abundances of the model proteins in metastable equilibrium were calculated. Also, here the chemical formulas of the proteins were normalized by number of residues, so that effects of varying protein length are removed.

In this study, the metastable equilibrium model results in deterministic predictions about the relative abundances of specific taxa. The mechanisms of community assembly are not explicitly considered, and this sets the metastable equilibrium model apart from process-based models such as neutral community models (NCM) [11], [12] that yield probability distribution functions, or, in a discrete case, counts of taxa for a bin of relative abundance. In contrast, the discrete outputs of the metastable equilibrium model are relative abundances of each specific taxon in the model. Therefore, the metastable equilibrium model is independent from, and might be used to complement, process-based models of microbial community assembly. Like the continuum NCM [11], [12], the metastable equilibrium model also has the inherent flexibility to reproduce any particular taxon distribution, but that outcome is dependent not on rates of birth, death and immigration (in the case of NCM) but on the relative Gibbs energies of the biomass, and on the temperature, pressure, and chemical potentials used to describe the physical-chemical environment. This versatility in modeling specific distributions is perhaps an advantage of the geochemically constrained metastable equilibrium approach that could complement more mechanistically based models in ecology.

Metastable equilibrium does not imply complete dominance of a single, fittest species, but rather a combination of taxa (in this case, phyla) coexisting at different abundances. Again, the deterministic chemical equilibrium model shares this characteristic with stochastic models of communities [31]. An implication of the metastable equilibrium model is that increased fitness, when quantified in reductionist terms as energetic cost of biomass synthesis, is not “lowest energy” but “right amount of energy” given the environmental conditions and relative abundance of the organism in the population.

## Conclusions

Observed phylum-level abundance profiles from BLAST classifications of metagenomically derived protein sequences in the Bison Pool metagenome were modeled using metastable equilibrium among model proteins for each phylum. Measurements of chemical activities of basis species were used in the calculations, except for 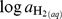, which was varied in order to minimize the distance between the calculated and observed assemblages. The major findings of this study are:

1. Along the outflow channel, there is an increase in the average oxidation state of carbon (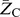) of the model proteins for major phyla. The range of values of 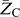, reflecting compositional differences among phyla, decreases at lower temperatures (Fig. 1).
2. A published revision of the standard-state Gibbs energy of the methionine sidechain group to a higher value, making it less stable, leads to relative stabilization of proteins with lower sulfur content, including those from the photosynthetic fringe (site 3). The appearance of site 3 on revised stability diagrams for model proteins based on functional annotations (Fig. 2) supports the notion of a progression of metastable equilibrium states along the the outflow channel.
3. The Gibbs energy of transformation between the metastable equilibrium assemblages of model proteins for phyla and the observed abundances, calculated using an expression based on chemical affinity (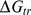), and using the information-theoretic difference (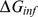), were found to give equivalent results when normalized chemical formulas are used. Optimal values of 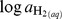 that minimize 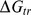 for each site were calculated. The results show that the lower-temperature sites are generally closer to metastable equilibrium (Figs. 3, 5).
4. Optimal values of 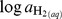 were found to decrease along the outflow channel, consistent with an increase in the oxidation potential of the hot-spring water as it cools and reacts with atmospheric gases and with metabolic products of the biofilms. The decrease in 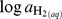 found in the current study is in the same direction, but more pronounced than values taken from a previously published equation that represents the relative stabilities of model proteins based on functional annotations (Fig. 4).
5. The overall resemblance of the model predictions to the observations indicates that more abundant organisms tend to be composed of proteins that have a relatively lower energy demand for formation at equal thermodynamic activities, and vice versa. The metastable equilibrium model underpredicts the relative abundances of Cyanobacteria and, to a lesser extent, Chloroflexi at sites 4 and 5 (Fig. 5). The deviation of the real system from the metastable equilibrium state is consistent with the onset of photosynthesis as an input of energy that is not available to the higher-temperature chemotrophic communities.

The approach described here is generally applicable to ecosystems where geochemical parameters as well as chemical compositions, relative abundances and standard Gibbs energies of the major biochemical constituents of organisms can be measured or estimated. Gibbs energy minimization is an established technique used for predictions of equilibrium mineralogy from the temperature, pressure and bulk composition of a rock. The present model extends those concepts to local Gibbs energy minimization and metastable equilibrium predictions of relative abundances of organisms in microbial ecosystems. Unlike predictions of minerals in rocks, the presence or absence of the organisms is not a feature of the metastable equilibrium model. Nevertheless, the construction of the metastable equilibrium model enables predictions linking geochemistry, biomolecular composition, and relative abundances of microbial phyla, and reveals an energetic basis for many of the observed patterns.

## Methods

### BLAST, Community Profile and Model Proteins

All microbial protein sequences in Reference Sequence (RefSeq) database release 57 (2013-01-08) were downloaded from NCBI (http://www.ncbi.nlm.nih.gov/refseq/). The number of sequences is 24,477,649 covering 7415 unique taxon identifiers. From this, a BLAST [20] version 2.2.24 database was constructed using formatdb. Amino acid sequences for inferred proteins in the Bison Pool metagenome, automatically produced by the IMG/M pipeline, were downloaded from the website of the Joint Genome Institute (JGI) (http://img.jgi.doe.gov/m). A search of matches of the metagenomic query sequences in the RefSeq target database was performed with the blastp program, using default E value (10.0) and similarity parameters. Using the generated blastp output files, the hits were filtered to keep only those with a similarity score of 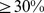 and E value of 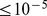. The number of hits filtered at this step was more affected by the E value cutoff than the similarity score cutoff. After these filtering steps, only the first database hit for each query sequence was kept. The resulting tabular BLAST output files are provided in Dataset S1.

In the blastp output files, there are hits to 98894 unique sequences in the RefSeq database. The identifiers (gi numbers) of these sequences are associated with taxonomic identifiers (taxid) in the file gi.taxid.txt in Dataset S2. The gi-taxid mappings were extracted from the file RefSeq-release57.catalog on the NCBI ftp site (ftp://ftp.ncbi.nih.gov/refseq/release/release-catalog, accessed on 2013-01-19). For each taxid, the phylum name was obtained from the names.dmp and nodes.dmp files that are part of the taxonomy data available on the NCBI ftp site (ftp://ftp.ncbi.nih.gov/pub/taxonomy/, accessed on 2013-01-15). Individual phyla whose sequence counts numbered at least 3% of the total number of BLAST hits at each site were considered to be the major phyla modeled in this study. The amino acid compositions of all sequences assigned to each major phylum were averaged to give the amino acid compositions of model proteins that are provided in the file bison_protein.csv in Dataset S2.

### Thermodynamic Conventions

We generalize the calculations of metastable equilibrium abundances in systems of proteins by writing, for each protein, a reaction to form one mole of the residue-normalized formula of the protein from the basis species 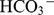, 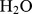, 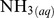, 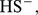 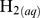 and 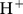. The reactions represent mass-balance constraints on possible transformations among proteins, each of which has a standard Gibbs energy that is also associated with the specific amino acid composition of the protein. All species are taken to be in the aqueous phase; the standard state corresponds to unit activity of the pure solvent (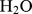), or unit activity of solute species (other than 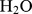) in a hypothetical one molal solution referenced to infinite dilution at any temperature and pressure.

A “residue formula” (or just “residue”) represents the length-normalized chemical formula of a protein. For example, the residue formula of a protein with a sequence length of 129 amino acids with chemical formula 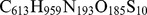 has a chemical formula of.


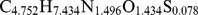
 (coefficients rounded to 3 decimal places) and a standard molal Gibbs energy (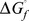) that is 1/129 that of the whole protein. It has been shown that mass-action equations for chemical reactions between residue formulas of proteins are consistent with metastable coexistence of proteins with comparable chemical activities rather than total predominance and either-or type behavior when non-normalized protein formulas are used [32].

The ionization states and standard Gibbs energies of the proteins in the reactions are estimated using amino acid group additivity for unfolded proteins [16] including contributions from ionizable sidechain and terminal groups whose degrees of ionization depend on temperature and pH. As noted elsewhere [16], [19], [32], the standard Gibbs energy changes of protein folding reactions are much smaller than the standard Gibbs energies of the proteins themselves, and tend to cancel in metastability calculations (where proteins occur on both sides of the transformation reactions), so the standard Gibbs energies of protein folding have a negligible, or at least secondary effect, in calculations of the relative chemical stabilities of proteins, and are not considered below. Recently updated values for the contributions of the methionine sidechain group ([Met]) [27] do, however, have a significant effect on the relative stabilities of proteins, and are used in the present study.

The standard Gibbs energies of the proteins and basis species at elevated temperatures and pressures are calculated using the revised [33] Helgeson-Kirkham-Flowers equation of state [34] for aqueous species, with thermodynamic data and equation-of-state parameters for the basis species taken from [35]–[37]. As used here, “standard Gibbs energy” refers to a specified temperature (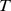) and pressure (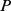) that are in general different from the reference temperature (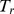) and pressure (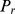) of 25°C and 1 bar. However, since all temperatures considered here are below 100°C, the value of 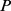 is always 1 bar. The activities of the basis species used in the calculations are 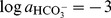, 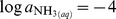, 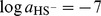, 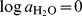, 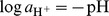 taken from Table 1, and 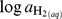 taken from Eq. (2) [19] or used as a fitting parameter to optimize the model.

### Chemical Affinity

For the 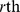 reaction at specified temperature and pressure, chemical affinity (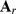) is calculated using
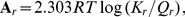
(3)where 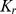 and 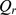 denote the equilibrium constant and activity quotient of the reaction, 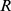 stands for the gas constant, and 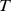 stands for temperature in kelvin. The symbol 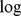 is used to indicate decimal (base 10) logarithms; the 2.303 is shorthand for the natural logarithm of 10. The value of 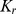 is calculated using
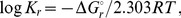
(4)where 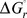 stands for the standard Gibbs energy change of the 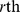 reaction at a specified pressure and temperature. The value of 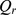 is calculated using
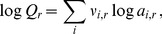
(5)where 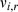 and 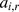 represent the stoichiometric reaction coefficient and the chemical activity of the 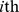 species in the 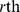 reaction, respectively, and the summation is over all species in the reaction.

From this point forward, we use 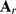 to denote the affinities of reactions written for the formation of one mole of the residue formula of a protein from the basis species, i.e. 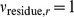. A specific example is described in detail further below. In this thermodynamic model for open systems, chemical transformations among residues take place at constant chemical potential of each basis species. In order to quantify the metastable equilibrium activities of residues and the Gibbs energies of transformation from metastable equilibrium to non-equilibrium assemblages, it it convenient to define 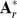 as
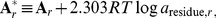
(6)where 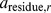 denotes the activity of the residue in the 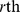 reaction. Because the formation reactions of the residues all have 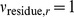, changing the value of 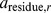 has no effect on the value of 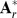, which can be seen by comparing Eq. (6) with Eqs. (3) and (5). The starred affinity 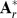 is “starved” of the activity of the residue; for a given residue it is a function only of temperature, pressure and chemical activities of the basis species. Where energetic properties at equal thermodynamic activities are discussed, it is in reference to 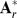 in Eq. (6).

### Boltzmann Distribution as a Metastable Chemical Equilibrium

The activities (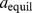) and degrees of formation (i.e. relative abundances expressed as mole fraction, 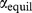), of the residue formulas in a metastable equilibrium assemblage of model proteins are calculated using
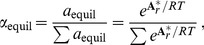
(7)where the summations are taken over all of the residues in the assemblage. Eq. (7) relates chemical affinities (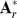) to relative abundances of residue formulas in a system in metastable equilibrium in the same way that a physical interpretation of the Boltzmann distribution relates energy levels (e.g. of electronic configurations of an atom) to occupancies. The summation of activities in the denominator of Eq. (7) is only valid if the activities are approximated as being equal to concentrations, i.e. activity coefficients of all species are equal to unity. For a given total activity of residues, the 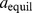 of each residue computed using Eq. (7) can be substituted into Eq. (3), with the result that all 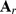 are equal, but not necessarily equal to zero; this satisfies the definition of metastable equilibrium [38].

The methods for computing the metastable equilibrium activities of the model protein residues can be summarized as 1) calculate the standard Gibbs energies of the formation reactions of the residues from the basis species, 2) combine the standard Gibbs energies with chemical activities of the basis species to calculate 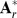 for each residue in the system, 3) use the Boltzmann distribution to calculate the degrees of formation (relative abundances) of the residues in metastable equilibrium. In our previous study [19], relative abundances of the residues were converted to relative abundances of the model proteins using the average lengths of the fragments. However, because the model proteins (in both the previous and present studies) were derived from fragmentary metagenomic sequences, no reliable length information is available. Therefore, in building the present model from fragmentary environmental sequence data, we now find it advisable to make no assumptions about the lengths of the proteins and instead refer only to relative abundances of residue formulas in metastable equilibrium.

### Energetic Distance from Equilibrium

In an open system, calculating the energetic difference between an assemblage of species in metastable equilibrium and any observed assemblage of chemical species must take account of the Gibbs energies of all reactions as they proceed under constant temperature, pressure, and chemical activities of basis species. In the present model the chemical species of interest are the residue formulas of the proteins. First consider the differential Gibbs energy of the system (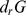) occurring with an increment of reaction progress for the 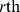 reaction (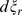):
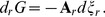
(8)


Eq. (8) is simply a rearrangement of the definition of chemical affinity [38]. As noted above, all formation reactions of residues are written with 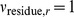. Recall that each formation reaction is uniquely written for a single residue, so the index 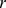 can be used either for residues or their formation reactions. Therefore,
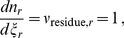
(9)where 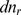 stands for the differential number of moles of the residue in the 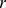
th formation reaction. Combining Eqs. (8) and (9) permits writing an expression for 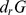 in terms of moles of residue formed (or destroyed) in place of the reaction progress variable:



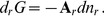
(10)The derivatives in Eq. (10) consist of extensive variables. By dividing both sides of Eq. (10) by 1 kg 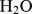, we can write
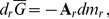
(11)where 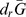 denotes the differential molal Gibbs energy of the system associated with net formation or destruction of the 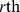 residue, and 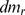 stands for the differential molality of the 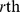 residue. In the limit of ideality,
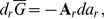
(12)where 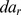 stands for the differential activity of the residue, which in an ideal solution containing 1 kg of 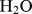 is equal to differential molality (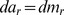). Incorporation of non-ideal interactions in this derivation would require additional terms for activity coefficients that are not considered at present.

If the initial activity of the 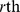 residue is given by 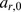 and the final activity after chemical transformation of the system by 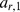, it follows that
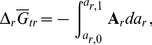
(13)where 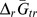 denotes the molal Gibbs energy change associated with net formation or destruction of the 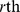 residue as a result of the chemical transformation of the system.

As written, Eq. (13) has no general analytical solution because 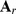 is a function of 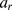 (Eqs. 3 and 5). Combining Eqs. (6) and (13) gives
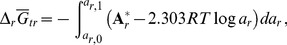
(14)in which 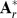 is independent of 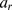, as noted above in relation to Eq. (6). Therefore, Eq. (14) can be integrated to write



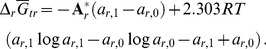
(15)The overall molal Gibbs energy of transformation of the system (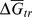) can then be calculated from
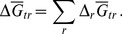
(16)


If the 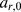 correspond to a metastable equilibrium assemblage, then we expect to find 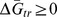 for any chemical transformation, i.e. to form an observed assemblage that is not in metastable equilibrium, taking place at constant 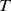, 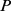, and chemical activities of the basis species.

In this study, the primary variable denoting the relative abundances of the model proteins and of the microbial phyla is not activity (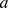) but instead fractional degree of formation (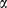). In all of the model computations, the total activity of protein residues is set equal to unity. If follows, then, that analogs of Eqs. (15–16) in terms of the observed and metastable equilibrium degrees of formation (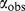 and 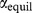, respectively) can be written as
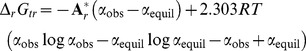
(17)and



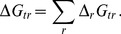
(18)Because of the dual conventions of unit activity coefficients and unit total activity of residues, the 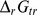 and 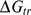 in Eqs. (17–18) are implicitly molal quantities, unlike their counterparts in Eqs. (15–16), which were explicitly derived for a solution containing one kg of 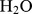.

### An Information-theoretic Measure of Distance from Metastable Equilibrium

The terms in Eq. (15) are related to work in a chemical-potential field (represented by 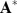) and to Gibbs energy of ideal mixing (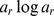 modified by 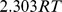) which combine to yield a measure of energetic difference between two states of an open reacting system. An information-theoretic measure known as the relative entropy [39], Kullback measure for the increment of information [40], or Kullback-Leibler divergence is
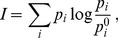
(19)where 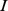 is the relative information difference between an initial probability distribution 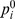 and a final probability distribution 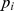. It has been noted that Eq. (19) is related to the difference in free energy between the states (e.g. [39]). An expression for the information-theoretic entropy of transition (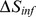), as applied to discrete states of a chemical system, results from substituting 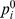 with the metastable equilibrium relative abundances of species (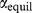) and 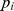 with the observed relative abundances of species (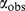):



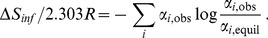
(20)The calculation of molar values of 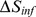 using Eq. (20) is provided for by the factor of 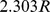, which includes the gas constant (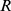) and a multiplier (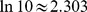) that when removed from the equation transforms the base 10 logarithm to a natural logarithm. If the transition is an ideal mixing process, so that the associated enthalpy of the transition is zero [41], the Gibbs energy of transition (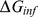) associated with the information-theoretic entropy difference can then be written as
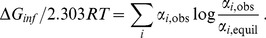
(21)


We found that calculations of 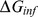 using Eq. (21) yield results that are numerically equal to the values of 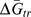 calculated using Eqs. (15–16), shown in the bottom row of Fig. 3. This finding is consistent with an equivalence between the information-theoretic concept of relative entropy and a thermodynamic entropy of transition accompanying chemical reactions when numbers of moles of species are conserved, which is the case in the model because the length-normalized formulas of proteins (“residue formulas”) are the reactants. However, a trial calculation for a different chemical system (homologous series of 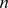-alkanes) having a variable number of moles of species through the transformation from metastable equilibrium to non-equilibrium, results in unequal values of 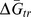 and 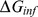. Further investigation may be required to understand the differences, but the implied consequence is that Eq. (21) only constitutes a measure of distance from metastable equilibrium in chemical systems where reactions between species conserve moles of species, and that Eqs. (15–16) are applicable in the more general case for chemical systems where reactions do not conserve total numbers of moles of species.

### Metastable Equilibrium Degrees of Formation: Example Calculation

An example of the steps in the metastable equilibrium calculation follows, and can be computed in software using the equil.example() function in mem-ramp.R (Dataset S2). The overall formation reaction from basis species for one mole of the length-normalized model protein (residue formula) for Aquificae at site 1 can be written as
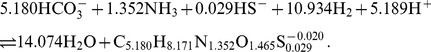
(22)


The residue formula of the protein is available in protein_table.csv in Dataset S2 along with the value of 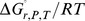 for this reaction (−193.29) computed at the temperature of site 1 (93.3°C). The activities of the basis species are taken to be 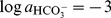, 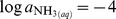, 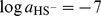, 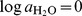 and, for site 1, 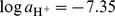, and 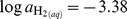; this value of 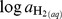 was found in this study to optimize the fit between the model and observed relative abundances (Table 1). Combining these values with Eqs. (5–6) yields 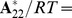 −28.306 for the residue formula for the model protein for Aquificae. Analogous calculations for the remaining model proteins for site 1 give values of 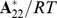 equal to −29.313, −30.267, −29.289, and −29.022, for the residue formulas for model proteins for Crenarchaeota, Proteobacteria, Deinococcus-Thermus and Firmicutes. Substituting these 5 values of 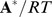 into Eq. (7) yields metastable equilibrium degrees of formation of the residue formulas (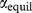) of 0.422 (Aquificae), 0.154 (Crenarchaeota), 0.059 (Proteobacteria), 0.158 (Deinococcus-Thermus) and 0.206 (Firmicutes). The value of 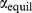 is highest for Aquificae, i.e. the model protein with the least negative value of 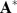, illustrating an inverse relationship between the metastable equilibrium abundance and the overall energy required to form the protein at equal thermodynamic activities.

As noted above, the computed relative abundances of the residue formulas are taken to be equal to those of the model proteins because accurate protein-length information is not available from fragmentary metagenomic sequences. Therefore, at site 1, using the value of 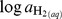 specified above, the model protein with the highest abundance in the metastable assemblage is that for Aquificae, and the model protein for Proteobacteria is the least abundant. These metastable equilibrium abundances are the same as those shown in Figs. 5a and 6b.

The relative abundances of the phyla at site 1 in the observed (BLAST) profile (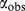), in the order shown in Table 2 and used in the example above, are 0.549, 0.198, 0.154, 0.058, and 0.041. Substitution into Eqs. (17–18) of these values of 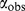, and of 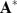 and 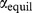 calculated above, gives a value of 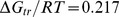. Likewise, substitution of 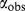 and 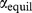 into Eq. (21) gives 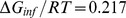.

### Source Code and Software Package

The thermodynamic calculations were performed using functions and data files that are part of the freely available CHNOSZ software package [32], version 1.0.1, for the R software environment [42]. The package is available for download from the Comprehensive R Archive Network (CRAN, http://cran.r-project.org/). The code used to construct the figures appearing in this paper is provided in the file mem-ramp.R as part of Dataset S2. The most recent release of the package, developed in conjunction with this study, includes updates to data files and functions used in the processing of the BLAST output and in the calculations of metastable equilibrium abundances. The similarity, E value, and maximum hits filtering were performed using the read.blast() function in CHNOSZ. Besides the BLAST processing functions, other features of the package that recently were modified include improvements in the functions used to compute the ionization states and standard molal properties of protein ionization, incorporation of updated methionine group contributions and properties of crystalline sidechain and protein backbone groups [27], and a function to calculate the Gibbs energy of transformation (Eqs. 15–16).

## Supporting Information

Figure S1 **Predominance diagrams.** Metastable equilibrium predominance diagrams for model proteins in different functional categories, as in Fig. 2, but with a more extensive set of model proteins, for comparison with Figure 6 of [19].
(PDF)

Dataset S1 **BLAST output.** Protein BLAST tabular output files listing first hit for each query sequence.
(ZIP)

Dataset S2
**Data files.** Data files and source code used in this study, including the files taxid_names.csv (names at different taxonomic levels for microbial taxa in RefSeq 57), prep.R (R source code, with functions to prepare data files), gi.taxid.txt (unique gi numbers and taxon identifiers (taxid) for all BLAST hits), bison_protein.csv (amino acid compositions of model proteins for the phyla), mem-ramp.R (R source code, with functions to create each of the figures), and protein_table.csv (model proteins, including information from Tables 2 and 3, and values of 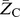 and 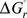). Note that prep.R contains functions to process data files that are not provided as supporting information (RefSeq and taxonomy files from NCBI and Bison Pool metagenome from JGI), but that all figures can be produced using the code in mem-ramp.R together with the provided data files.(ZIP)
