# Supplementary material for: A Metastable Equilibrium Model for the Relative Abundances of Microbial Phyla in a Hot Spring
Source: PLoS One. 2013 Sep 2;8(9):e72395. doi: 10.1371/journal.pone.0072395 (PMC3759468; doi:10.1371/journal.pone.0072395)
Supplement: Figure S1 — Predominance diagrams. Metastable equilibrium predominance diagrams for model proteins in different functional categories, as in Fig. 2, but with a more extensive set of model proteins, for comparison with Figure 6 of [19]. (PDF) [file pone.0072395.s001.pdf]

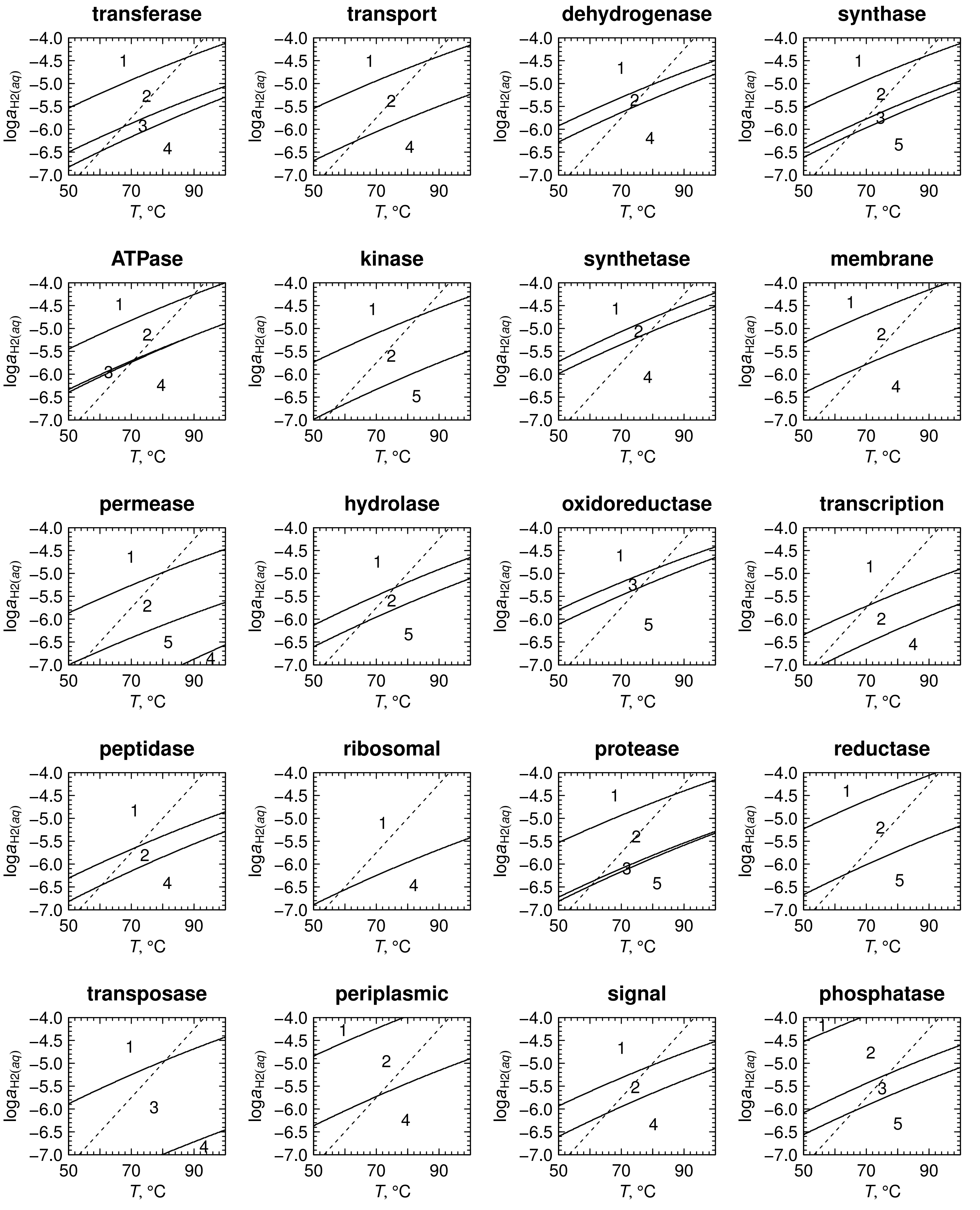

transferase
1
2
3
4
transport
1
2
4
dehydrogenase
1
2
4
synthase
1
2
3
5
ATPase
1
2
3
4
kinase
1
2
5
synthetase
1
2
4
membrane
1
2
4
permease
1
2
5
4
hydrolase
1
2
5
oxidoreductase
1
3
5
transcription
1
2
4
peptidase
1
2
4
ribosomal
1
4
protease
1
2
3
5
reductase
1
2
5
transposase
1
3
4
periplasmic
1
2
4
signal
1
2
4
phosphatase
1
2
3
5
−4.0
−4.5
−5.0
−5.5
−6.0
−6.5
−7.0
$\log a_{H2(aq)}$
50
70
90
T, °C
